# Metabolic and microvascular function assessed using near‐infrared spectroscopy with vascular occlusion in women: age differences and reliability

**DOI:** 10.1113/EP090540

**Published:** 2022-11-24

**Authors:** Emily M. Rogers, Nile F. Banks, Nathaniel D. M. Jenkins

**Affiliations:** ^1^ Integrative Laboratory of Applied Physiology and Lifestyle Medicine University of Iowa Iowa City IA USA; ^2^ Abboud Cardiovascular Research Center University of Iowa Iowa City IA USA

**Keywords:** cardiovascular disease, flow‐mediated dilatation, microvascular, NIRS‐VOT, reactive hyperaemia, reperfusion, responsive hyperaemia, women

## Abstract

We investigated the test–retest reliability of, and age‐related differences in, markers of skeletal muscle metabolism and microvascular function derived from the near‐infrared spectroscopy with vascular occlusion test (NIRS‐VOT) in younger women (YW) and middle‐aged and older women (MAOW). Seventeen YW (age 23 ± 4 years) and 17 MAOW (age 59 ± 8 years) completed this study. Participants completed identical experimental visits separated by ∼4 weeks during which the NIRS‐VOT was used to quantify the occlusion slope, minimum and maximum tissue saturation, ischaemic index, reperfusion magnitude, the reperfusion and 10‐s reperfusion slopes (slope 2 and slope 2_10‐s_), time to max tissue saturation, and area under the reperfusion curve using the local tissue oxygen saturation signal. Except for slope 2_10‐s_ (intraclass correlation coefficient (ICC) = 0.37; coefficient of variation (CV) = 31%), time to max tissue saturation (ICC = 0.21), and ischaemic index (ICC = 0.37) for MAOW, all of the NIRS variables demonstrated good to excellent relative reliability for the YW (ICCs = 0.74–0.86) and the MAOW (ICCs = 0.51–0.87), with CVs of 2–21% and 2–22%, respectively. The occlusion slope was significantly lower, indicating accelerated deoxygenation, while maximum tissue saturation, reperfusion magnitude, and ischaemic index were significantly higher in YW versus MAOW. No other group differences were found. In conclusion, our data support the use of the NIRS‐VOT as a simple, reliable, non‐invasive technique for the assessment of peripheral skeletal muscle metabolism and microvascular function in women, with the reliability being generally greater in YW versus MAOW. Further, our data suggest that ageing is associated with lower skeletal muscle metabolism and microvascular hyperaemic responsiveness in women.

## Abstract (highlights)

- **What is the central question of this study?**
  Can the near‐infrared spectroscopy with vascular occlusion test (NIRS‐VOT) reliably measure skeletal muscle metabolic and microvascular function in women?
- **What is the main finding and its importance?**
  The NIRS‐VOT can be used as a reliable technique for the assessment of skeletal muscle metabolism and microvascular function in women, with reliability being generally greater in younger women. These findings have important implications for the planning and development of future studies employing the NIRS‐VOT in women, and provide insights into the effects of age on these parameters in women specifically.

## 1 INTRODUCTION

Non‐invasive tests that employ transient ischaemia in order to provoke vasodilator responses to assess vascular endothelial function have been particularly useful prognostic indicators of cardiovascular disease (CVD) risk (Green et al., 2011; Huang et al., 2007; Ishibashi et al., 2006; Paine et al., 2016), the foremost of which is the flow‐mediated dilatation (FMD) technique. Over the last several years, a non‐invasive test employing the use of near‐infrared spectroscopy (NIRS), known as the NIRS with vascular occlusion test (NIRS‐VOT), has been suggested as a non‐invasive technique for the assessment of skeletal muscle microvascular function in clinical medicine and exercise physiology settings (Tucker et al., 2020; Vardi & Nini, 2008). Importantly, when used in conjunction with vascular occlusion, analysis of the NIRS tissue oxygen saturation ($S_{{\mathrm{tO}}_{2}}$) signal provides dynamic measurements of both tissue oxygen utilization during ischaemia and the subsequent hyperaemic response (Rosenberry & Nelson, 2020). Specifically, analysis of the rate of decline in $S_{{\mathrm{tO}}_{2}}$ during ischaemia provides important information regarding metabolic health, where greater muscle oxidative function is associated with steeper reductions in $S_{{\mathrm{tO}}_{2}}$ (Chung et al., 2018; Rosenberry et al., 2018; Ryan et al., 2013). Analysis of the hyperaemic magnitude and rate of reperfusion is thought to primarily reflect microvascular flow, which has been shown to demonstrate a strong relationship with post‐occlusive conduit artery blood flow (i.e., reactive hyperaemia) (Bopp et al., 2014). Consequently, the NIRS‐VOT has been used in several studies as a tool to assess skeletal muscle metabolic and microvascular function, and has been shown to be sensitive to changes in altitude (Martin et al., 2013), altered microcirculatory function in sepsis (Stout & Crawford, 1988), the presence of CVD risk factors (de Oliveira et al., 2019; Rosenberry et al., 2018), aerobic fitness levels, sex differences (Rasica et al., 2022) and body fatness (Soares & Murias, 2018).

Despite the increasing frequency with which this technique has been used, while a few studies have examined the reliability of the parameters assessed using the NIRS‐VOT (Gayda et al., 2015; Iannetta et al., 2019; Kragelj et al., 2000; Lacroix et al., 2012; McLay, Nederveen, et al., 2016), none of these have examined the reliability in women specifically. Further, only one study has explored age‐related differences in microvascular function variables in women using the NIRS‐VOT (Horiuchi & Okita, 2020). Not only are studies such as these necessary for understanding the utility of the NIRS‐VOT in women, but also because microvascular dysfunction may precede pathological changes in the macrovasculature (Jung et al., 2013), and thus non‐invasive techniques that are sensitive to decrements in microvascular function are particularly useful for early detection of CVD risk.

Therefore, the purpose of this study was to examine the relative and absolute reliability of commonly reported parameters that reflect skeletal muscle metabolic and microvascular function using the NIRS‐VOT in women. A secondary purpose was to assess age‐related differences in these NIRS‐VOT‐derived variables in women. We hypothesized that (1) the NIRS‐VOT variables would display sufficient reliability, as indicated by intraclass correlation coefficients (ICCs) ≥0.60 and low coefficients of variation in both groups and (2) that there would be age‐related differences among these variables, such that markers of metabolic and microvascular function would be greater in younger women (YW) than middle‐aged and older women (MAOW).

## 2 METHODS

### 2.1 Ethical approval

Thirty‐five females were enrolled in and completed this study. All participants self‐identified as women. The study was conducted in accordance with the *Declaration of Helsinki*, except for registration in a database. Informed consent was obtained from each participant in writing. Participants were recruited at two different universities in the southwest and mid‐west regions of the USA, due to interruption via the COVID‐19 pandemic and the investigative team moving universities. This investigation was approved by, and carried out in accordance with, the universities’ Institutional Review Boards for the protection of human subjects (IRB Approval nos ED‐19‐50‐STW and 202104167, approved 30 May 2019 and 13 May 2021, respectively).

### 2.2 Study design

Data from only 34 participants were used in analyses due to the inability to obtain a quality NIRS signal in one participant. Participants were eligible for this study if they were female, between the ages of 18 and 35 years or 45 and 75 years and had a body mass index (BMI) < 40 kg/m^2^. Participants aged 18–35 years were defined as young women (YW; *n* = 17), whereas participants aged 45–75 years were defined as MAOW (*n* = 17). Fourteen of the MAOW were postmenopausal, three were premenopausal and none were perimenopausal. Participants were not excluded for being hypertensive (having a blood pressure (BP) of >130/>80), having diabetes, or for taking antihypertensive medications, statins or diabetes medication.

To examine the reliability of the NIRS‐VOT in YW and MAOW, participants visited the lab on three separate occasions, once for screening and twice to undergo physiological testing. During the first visit (T0), participants were screened, signed an informed consent form, completed a health history questionnaire, were enrolled if eligible, and had their height and weight measured. The participants then returned to the lab following a 10‐h fast for experimental visits 1 (T1) and 2 (T2). Participants were asked to refrain from exercise, caffeine, alcohol, and over‐the‐counter medication for 24 h prior to each visit, and were also instructed to maintain prescription medication use as normal prior to each visit. All testing was performed between 06.00 and 10.00 h, except for one YW participant who began both experimental visits at 13.00 h. Whereas prior studies have provided 2‐week periods between test–retest visits (Iannetta et al., 2019), we tested participants 28 ± 5 days apart in an attempt to test women during the same phase of their menstrual cycle (Stanhewicz & Wong, 2020; Wenner & Stachenfeld, 2020). During T1 and T2, the NIRS‐VOT was performed to assess indices of skeletal muscle metabolic and microvascular function. In addition, analysis of fasting metabolic markers, FMD, BP and body composition tests was performed to provide information on the general metabolic and vascular health of this sample.

### 2.3 Near‐infrared spectroscopy with vascular occlusion test

**FIGURE 1:**
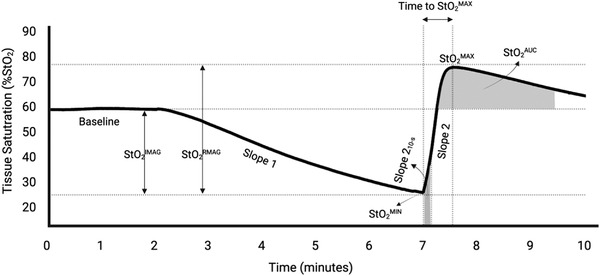
Tissue oxygen saturation ($S_{{\mathrm{tO}}_{2}}$) signal during a near‐infrared spectroscopy with vascular occlusion test (NIRS‐VOT). Cuff inflation occurred at 2 min and deflation occurred at 7 min to induce ischaemia and hyperaemia, respectively. $S_{{\mathrm{tO}}_{2}\mathrm{imag}}$, magnitude of ischaemia; $S_{{\mathrm{tO}}_{2}\mathrm{rmag}}$, reperfusion magnitude; slope 1, desaturation rate; $S_{{\mathrm{tO}}_{2}\min}$, minimum saturation value observed; slope 2, reperfusion rate; slope 2_10‐s_, oxygen reperfusion rate during the 10‐s window immediately post cuff deflation; $S_{{\mathrm{tO}}_{2}\max}$, maximum saturation value observed; time to max $S_{{\mathrm{tO}}_{2}}$, difference in time from $S_{{\mathrm{tO}}_{2}\min}$ to $S_{{\mathrm{tO}}_{2}\max}$; $S_{{\mathrm{tO}}_{2}\mathrm{AUC}}$, 2‐min reperfusion area under the curve after signal returned to baseline after cuff release

The NIRS‐VOT was used to assess local tissue $S_{{\mathrm{tO}}_{2}}$ during occlusion and reperfusion. A NIRS device (PortaMon, Artinis Medical Systems, Einsteinweg, The Netherlands) was placed on the muscle belly of the flexor digitorum superficialis on the arm and environmental light interference was eliminated using an optically dense, black vinyl sheet and a flexible bandage wrap. Elastic athletic tape was also placed around the device to further minimize movement. A cuff was then placed on the upper arm and inflated to 240 mmHg for 5 min to induce ischaemia before being suddenly released to induce hyperaemia. The NIRS device was used to continuously collect local $S_{{\mathrm{tO}}_{2}}$ using infrared light for the 2 min prior to cuff inflation, during the 5‐min occlusion period, and during the 3 min following cuff release, as previously described (McLay, Fontana, et al., 2016). NIRS data were collected at an output frequency of 10 Hz, recorded on a personal computer, and then processed offline using custom‐written LabVIEW software (v. 18, National Instruments, Austin, TX, USA) in accordance with the methods described previously (de Oliveira et al., 2019; McLay, Fontana, et al., 2016; Rosenberry et al., 2018; Soares & Murias, 2018; Soares et al., 2017). In brief, baseline $S_{{\mathrm{tO}}_{2}}$ represented the level of $S_{{\mathrm{tO}}_{2}}$ before cuff inflation. After cuff inflation, the desaturation rate (i.e., tissue oxygen extraction rate) was quantified as the downslope of $S_{{\mathrm{tO}}_{2}}$ and referred to as slope 1. The minimum $S_{{\mathrm{tO}}_{2}}$ ($S_{{\mathrm{tO}}_{2}\min}$) was calculated as the lowest value recorded during ischaemia. The magnitude of tissue ischaemia ($S_{{\mathrm{tO}}_{2}\mathrm{imag}}$) was calculated as the difference between baseline $S_{{\mathrm{tO}}_{2}}$ and $S_{{\mathrm{tO}}_{2}\min}$. The $S_{{\mathrm{tO}}_{2}}$ reperfusion slope was used to analyse microvascular reactivity and was quantified as the entire upslope of the $S_{{\mathrm{tO}}_{2}}$ signal immediately post‐cuff deflation until peak reperfusion (slope 2) and as the upslope of the 10 s window of the $S_{{\mathrm{tO}}_{2}}$ signal immediately post‐cuff deflation (slope 2_10‐s_). The reperfusion magnitude ($S_{{\mathrm{tO}}_{2}\mathrm{rmag}}$) was quantified as the difference between the $S_{{\mathrm{tO}}_{2}\min}$ and the maximum observed $S_{{\mathrm{tO}}_{2}}$ value ($S_{{\mathrm{tO}}_{2}\max}$) during reperfusion. Time to max $S_{{\mathrm{tO}}_{2}}$ was calculated as the difference in time (s) from $S_{{\mathrm{tO}}_{2}\min}$ to $S_{{\mathrm{tO}}_{2}\max}$. The reperfusion area under the curve ($S_{{\mathrm{tO}}_{2}\mathrm{AUC}}$) was calculated as the integrated area under the $S_{{\mathrm{tO}}_{2}}$ signal for a 2‐min time period after the signal returned to baseline following cuff release, as described previously (Soares & Murias, 2018) (Figure 1).

### 2.4 Flow‐mediated dilatation

Participants’ FMD was measured to assess endothelial function as described previously (Corretti et al., 2002) to characterize participants’ conduit artery function and in order to provide further context for the interpretation of any age‐related differences in NIRS‐VOT‐derived outcomes. The FMD measurement was performed on the arm opposite to that used for the NIRS‐VOT to avoid any effects of the previous 5‐min occlusion period on baseline blood flow, as these tests were performed back‐to‐back. In brief, the participant's arm was extended and immobilized by placing it in a prone positioning arm cradle (Health Products Express Inc., Boston, MA, USA). After lying quietly in a climate‐controlled room for 15 min, the brachial artery was imaged longitudinally with a portable brightness mode ultrasound device using a 12‐MHz linear transducer. Once a clear image of the artery was obtained, the probe was stabilized with a stereotactic probe holder (Single Axis Probe Holder, Quipu, Pisa, Italy). A BP cuff was inflated to 240 mmHg on the lower arm for 5 min to induce ischaemia before being suddenly released to induce hyperaemia. The brachial artery was visualized and recorded, using a high‐definition video capture device (AV.io HD, Epiphan Video, Palo Alto, CA, USA), continuously for the 2 min prior to cuff inflation, the 5‐min occlusion period and during the 3 min following cuff release (i.e., a total of 10 min). The recorded videos were then processed offline using continuous wall‐tracking software (FMD Studio, Quipu). The FMD% was calculated as the increase from baseline to peak artery diameter, expressed relative to the baseline diameter (%). One trained ultrasonographer performed all FMD tests and analyses. Recent coefficients of variation for trial‐to‐trial reliability for brachial artery baseline diameter, diameter change, and relative FMD in our laboratory are 2.6, 3.9 and 5.3%, respectively.

### 2.5 Blood collection and analysis

A small sample of capillary blood was taken from each participant during T1 and T2 using a 23‐gauge, push‐button safety lancet (McKesson, Irving, TX, USA). The participant's finger was cleaned with an alcohol wipe, pricked, and the first drop of blood removed, before a capillary tube (Alere Cholestech LDX Capillary Tubes 40 μl; Alere, San Diego, CA, USA) was used to extract the blood sample for analyses. Fasting metabolic markers, including high‐density lipoprotein cholesterol (HDL‐C), low‐density lipoprotein cholesterol (LDL‐C), total cholesterol (Total‐C), triglycerides (TG), and blood glucose were then assessed using a Cholestech 10‐990 LDX Cassette and Cholestech LDX analyser (Alere). If TG values were below the detectable limit (<45 mg/dl), a value of 44 mg/dl was used. The coefficients of variation for all markers were 2–6%.

### 2.6 Blood pressure

An automatic BP monitor (Omron Healthcare, Bannockburn, IL, USA) was placed on each participant's right arm. The participants sat quietly for 5 min prior to the BP assessment. Three assessments were performed, and the average of the two closest BP readings was recorded. Mean arterial pressure (MAP) was calculated as previously described (DeMers & Wachs, 2022) and used for analysis.

### 2.7 Framingham risk scores

The Framingham risk calculator was used to assess vascular age (F_VA_) and 10‐year risk (%). The risk score is based on age, systolic BP, HDL‐C, total‐C, smoking status, and the presence of diabetes and hypertension (D'Agostino et al., 2008).

### 2.8 Body composition

A Seca 514 medical body composition device (Seca, Hamburg, Germany) was used to assess participants’ body composition using multifrequency bioelectrical impedance analysis as previously described (Banks et al., 2021; Raeder et al., 2018). Participants were euhydrated and fasted for 10 h before body composition assessment.

### 2.9 Lifestyle controls

Participants were asked to record their food intake, including any calorie‐containing beverages, for two weekdays and one weekend day prior to T1 and T2, to assess their habitual dietary intake. Participants brought completed food logs with them to both of these visits. They were asked to give specific detail on brand names, serving sizes and preparation methods, and to enter food into their food log immediately after consumption. Dietary information was transferred into the ESHA Food Processor^®^ nutrition analysis Software (https://www.esha.com, ESHA Research, Oak Brook, IL, USA), which calculated absolute calories consumed (kcal) for each day. The average of the 3 days was taken for each time point and used later for analyses. Habitual physical activity levels were assessed using an International Physical Activity Questionnaire Long Last 7 days Format (IPAQ) at both T1 and T2. Metabolic equivalent task (MET) minutes were calculated from this questionnaire and examined to ensure similar physical activity levels for the week leading up to each visit (Hagstromer et al., 2006).

### 2.10 Statistical analyses

#### 2.10.1 A priori sample size determination

The sample size needed to estimate the value of ICCs was determined using the formula provided by Bujang and Baharum (2017), which was derived from Walter et al. (1998) and Winer (1962). Based on our primary hypothesis, we calculated the sample size needed to test whether the ICC was different from 0 and expected to be equal to 0.6 with a prespecified power (β) of 0.8 and 0.9, an a priori α of 0.05, and two measurement time points. We determined that between 15 (β = 0.8) and 20 (β = 0.9) participants per group were needed to test our primary hypothesis. Thus, 17 participants were recruited in each group, although data from only 14 YW and 16 MAOW are included in the reliability statistics herein due to signal artifact or software crashes during data collection that prohibited analyses from at least one experimental visit.

#### 2.10.2 Reliability

Both the YW and MAOW group means from T1 and T2 were compared for systematic variability using one‐way repeated measures analyses of variance (ANOVAs). Using these same ANOVA tables, ICCs were calculated using model 2,1 (ICC_2,1_) to quantify relative test–retest reliability. We utilized this model because ICC_2,1_ is a random factor model that includes both random and systematic factors in the denominator. Consequently, ICC_2,1_ can be generalized to outside raters and laboratories (Jenkins, Palmer, et al., 2014; Weir, 2005). The 95% confidence interval (95% CI) for each ICC was also calculated as previously described (Jenkins & Cramer, 2017; Jenkins, Buckner, et al., 2014; Shrout & Fleiss, 1979), in order to test the null hypothesis that each ICC was equal to zero. To promote qualitative interpretation of ICCs, the following schema was used: <0.5 indicated poor, 0.5–0.75 indicated moderate, 0.75–0.9 indicated good, and >0.9 indicated excellent relative reliability (Koo & Li, 2016). To measure absolute reliability between T1 and T2 for all dependent variables, the standard error of measurement (SEM) was calculated as follows (Hopkins, 2000; Weir, 2005):

(1) $$\mathrm{SEM}=\sqrt{{\mathrm{MS}}_{E}}$$

where MS_E_ was the mean square error term from the ANOVA table. In addition, the SEM was standardized as the coefficient of variation (CV) using the following equation (Hopkins, 2000):

(2) $$\mathrm{CV}=\frac{\mathrm{SEM}}{\mathrm{Grand}\;\mathrm{mean}}\times 100$$

#### 2.10.3 Group differences

Age comparisons (YW vs. MAOW) were made for baseline characteristics and NIRS‐derived metabolic and microvascular function indices. These comparisons were carried out on the average of each dependent variable at T1 and T2 using an independent‐samples Student's *t*‐test. Associations between age group and prevalence of medications taken, disease state (diabetes and hypertension) and postmenopausal status were determined using the chi‐square test of association. Where assumptions of this test were not violated (i.e., where all expected cell frequencies were greater than 5), a significant association was determined using Pearson's chi‐square asymptotic significance (two‐sided) value. If assumptions of this test were violated, Fisher's exact test exact significance (two‐sided) value was used to determine a significant association (Blalock, 1972). Pearson's product‐moment correlation coefficient was calculated to assess associations between FMD% and $S_{{\mathrm{tO}}_{2}\max}$, FMD% and $S_{{\mathrm{tO}}_{2}\mathrm{imag}}$, and Slope 2 and $S_{{\mathrm{tO}}_{2}\mathrm{imag}}$ using the grand mean for each variable from the T1 and T2 visits. Associations were examined across the entire sample, as well as within the MAOW and YW groups individually.

The a priori type I error rate was set at 5% for all analyses, which were performed using GraphPad Prism (version 8.0.0, GraphPad Software Inc., San Diego, CA, USA), Microsoft Excel 2019 (version 16.54), and IBM SPSS Statistics (version 28, IBM Corp., Armonk, NY, USA).

## 3 RESULTS

There were no significant differences observed in kcals, grams of fat, protein, or carbohydrates consumed or physical activity levels (MET‐minutes) in the days leading up to T1 and T2 for either group (all *P* > 0.053). Participant baseline characteristics for each group are shown in Table 1. In general, and as expected, MAOW displayed greater CVD risk factors than YW.

**TABLE 1:** Baseline characteristics for young versus middle‐aged and older women

Characteristic	YW (*n* = 17)	MAOW (*n* = 17)	*P*‐value	Effect size
Age (years)	23 ± 4	59 ± 8	**<0.0001**	**5.66**
Weight (kg)	64 ± 12	69 ± 11	0.200	
Height (cm)	167 ± 8	164 ± 6	0.200	
BMI (kg/m^2^)	23 ± 3	26 ± 4	**0.020**	
Kcal/day	1848 ± 463	1729 ± 319	0.390	
MET‐minutes/week	4702 ± 3788	3229 ± 1659	0.152	
SBP (mmHg)	107 ± 12	124 ± 21	**0.020**	**1.00**
DBP (mmHg)	73 ± 9	77 ± 12	0.276	
BF (%)	28 ± 7	37 ± 8	**0.001**	**1.15**
Blood glucose (mg/dl)	86 ± 8	91 ± 11	0.071	
Total cholesterol (mg/dl)	161 ± 27	188 ± 37	**0.010**	**0.84**
LDL‐C (mg/dl)	93 ± 25	107 ± 33	0.093	
HDL‐C (mg/dl)	49 ± 13	60 ± 13	**0.008**	**0.88**
Triglycerides (mg/dl)	93 ± 49	108 ± 50	0.187	
Framingham 10‐year risk (%)	0.7 ± 0.3	7.4 ± 6.4	**<0.0001**	**1.10**
Framingham vascular age (years)	22 ± 4	57 ± 19	**<0.0001**	**2.55**
Hypertensive (*n*)	1	6	0.085^FE^	
Blood pressure meds (*n*)	0	5	**0.044^FE^**	**0.415**
Statins (*n*)	0	3	0.227^FE^	
Diabetic (*n*)	0	1	0.500^FE^	
Metformin (*n*)	0	1	1.000^FE^	
Birth control (*n*)	9	1	**0.003^CS^**	**0.516**
Postmenopausal (*n*)	0	14	**<0.0001^CS^**	**0.837**

Values are calculated as the mean from T1 and T2 for each participant and are presented as group means ± SD. Independent samples *t*‐test or chi square/Fisher's exact test was performed to identify group differences, and resulting *P*‐values are displayed here with values shown in bold indicating statistically significant differences between groups. CS, chi‐square test was run; FE, Fisher's exact test was run. Effect sizes are presented where appropriate.

Abbreviations: BF%, body fat percentage; BMI, body mass index; DBP, diastolic blood pressure; MAOW, middle‐aged and older women; MET‐minutes, metabolic equivalent minutes; SBP, systolic blood pressure; YW, younger women.

### 3.1 Test–retest reliability of NIRS‐derived variables

The test–retest reliability statistics for the NIRS‐VOT parameters in the YW, MAOW and across the entire sample are displayed in Tables 2, 3, 4, respectively. All variables displayed moderate‐to‐good relative reliability in the YW (ICC_2,1_ ≥ 0.74) and MAOW (ICC_2,1_ ≥ 0.51), with the exceptions of Slope 2_10‐s_, time to max $S_{{\mathrm{tO}}_{2}}$ and $S_{{\mathrm{tO}}_{2}\mathrm{imag}}$ in the MAOW group. These variables displayed poor relative reliability (ICC_2,1_ = 0.37, 0.21 and 0.37, respectively). The absolute reliability (CV%) ranged from 2% to 21% for YW and 2% to 31% for MAOW.

**TABLE 2:** Test–retest reliability statistics for the variables derived from the near‐infrared spectroscopy with vascular occlusion test in younger women (*n* = 14)

	T1 mean ± SD	T2 mean ± SD	Grand mean ± SD	MS_B_	MS_B_ (%)	ICC_2,1_ (95% CI)	SEM	CV (%)	*P*‐value
Slope 1 (%/s)	−0.121 ± 0.031	−0.126 ± 0.034	−0.123 ± 0.030	0.002	1.6	0.753 (0.401, 0.913)	0.02	13.2	0.380
$S_{{\mathrm{tO}}_{2}\min}$ (%$S_{{\mathrm{tO}}_{2}}$)	28.7 ± 13.1	26.2 ± 14.7	27.5 ± 13.4	356.7	1297	0.839 (0.583, 0.945)	5.5	20.1	0.247
$S_{{\mathrm{tO}}_{2}\mathrm{imag}}$ (%$S_{{\mathrm{tO}}_{2}}$)	34.4 ± 9.2	35.3 ± 9.7	34.8 ± 8.9	157.9	453	0.768 (0.420, 0.919)	4.7	13.4	0.620
$S_{{\mathrm{tO}}_{2}\max}$ (%$S_{{\mathrm{tO}}_{2}}$)	76.6 ± 4.3	75.1 ± 5.3	75.9 ± 4.6	42.9	57	0.828 (0.513, 0.943)	1.8	2.4	0.060
Slope 2 (%/s)	1.068 ± 0.41	1.082 ± 0.44	1.075 ± 0.40	0.327	30	0.793 (0.465, 0.929)	0.2	18.6	0.851
Slope 2_10‐s_ (%/s)	1.484 ± 0.68	1.454 ± 0.70	1.469 ± 0.65	0.855	58	0.807 (0.496, 0.934)	0.31	21.2	0.797
$S_{{\mathrm{tO}}_{2}\mathrm{rmag}}$ (%$S_{{\mathrm{tO}}_{2}}$)	47.8 ± 11.4	48.9 ± 12.4	48.4 ± 11.2	251.9	520	0.779 (0.442, 0.923)	5.7	11.8	0.626
Time to max $S_{{\mathrm{tO}}_{2}}$ (s)	48.4 ± 12.8	48.7 ± 13.6	48.6 ± 12.7	321.3	661	0.856 (0.608, 0.952)	5.2	10.6	0.906
$S_{{\mathrm{tO}}_{2}\mathrm{AUC}}$ (%/s)	1223.9 ± 316.1	1184.5 ± 393.5	1204.2 ± 331.9	220299.4	18294	0.739 (0.364, 0.908)	185.6	15.4	0.584

Abbreviations: T1, visit 1; T2, visit 2; grand mean, mean from T1 and T2; MS_B_, mean square between; ICC, intraclass correlation coefficient; SEM, standard error of the measurement; CV, coefficient of variation; *P*‐value, type‐I error rate for the one‐way ANOVA used to assess systematic variability; slope 1, oxygen desaturation rate; $S_{{\mathrm{tO}}_{2}\min}$, minimum oxygen saturation observed; $S_{{\mathrm{tO}}_{2}\mathrm{imag}}$, magnitude of ischaemia; $S_{{\mathrm{tO}}_{2}\max}$, maximum oxygen saturation observed; slope 2, oxygen reperfusion rate; slope 2_10‐s_, oxygen reperfusion rate during the 10‐s window immediately post‐cuff deflation; $S_{{\mathrm{tO}}_{2}\mathrm{rmag}}$, reperfusion magnitude of oxygen saturation; time to max $S_{{\mathrm{tO}}_{2}}$, difference in time from $S_{{\mathrm{tO}}_{2}\min}$ to $S_{{\mathrm{tO}}_{2}\max}$; $S_{{\mathrm{tO}}_{2}\mathrm{AUC}}$, 2‐min reperfusion area under the curve after signal returned to baseline after cuff release.

**TABLE 3:** Test–retest reliability statistics for the variables derived from the near‐infrared spectroscopy with vascular occlusion test in middle‐aged and older women (*n* = 16)

	T1 mean ± SD	T2 mean ± SD	Grand mean ± SD	MS_B_	MS_B_ (%)	ICC_2,1_ (95% CI)	SEM	CV (%)	*P‐*value
Slope 1 (%/s)	−0.091 ± 0.028	−0.999 ± 0.029	−0.095 ± 0.025	0.001	1.1	0.512 (0.053, 0.796)	0.02	21.0	0.317
$S_{{\mathrm{tO}}_{2}\min}$ (%$S_{{\mathrm{tO}}_{2}}$)	34.2 ± 11.0	33.0 ± 9.0	33.6 ± 8.8	154.5	460	0.546 (0.078, 0.815)	6.9	20.4	0.625
$S_{{\mathrm{tO}}_{2}\mathrm{imag}}$ (%$S_{{\mathrm{tO}}_{2}}$)	26.1 ± 7.3	27.9 ± 7.6	27.0 ± 6.1	75.6	280	0.374 (−0.132, 0.725)	5.9	21.8	0.417
$S_{{\mathrm{tO}}_{2}\max}$ (%$S_{{\mathrm{tO}}_{2}}$)	71.9 ± 3.9	72.1 ± 3.9	72.0 ± 3.8	28.8	40	0.872 (0.672, 0.953)	1.4	2.0	0.656
Slope 2 (%/s)	1.000 ± 0.38	1.007 ± 0.24	1.003 ± 0.28	0.155	15	0.589 (0.133, 0.836)	0.2	20.4	0.932
Slope 2_10‐s_ (%/s)	1.445 ± 0.48	1.392 ± 0.60	1.393 ± 0.4	0.386	28	0.372 (−0.142, 0.726)	0.4	30.5	0.498
$S_{{\mathrm{tO}}_{2}\mathrm{rmag}}$ (%$S_{{\mathrm{tO}}_{2}}$)	37.7 ± 10.7	39.1 ± 9.2	38.4 ± 8.8	154.4	402	0.561 (0.104, 0.822)	6.7	17.4	0.551
Time to max $S_{{\mathrm{tO}}_{2}}$ (s)	39.3 ± 7.6	39.6 ± 7.1	39.5 ± 5.7	64.8	164	0.209 (−0.341, 0.636)	6.6	16.7	0.899
$S_{{\mathrm{tO}}_{2}\mathrm{AUC}}$ (%/s)	1019.4 ± 399.8	976.6 ± 303.4	998.0 ± 319.1	203666.7	20407	0.628 (0.205, 0.852)	219.6	22.0	0.589

Bolded ICC values indicate significant relative reliability was not demonstrated.

Abbreviations: T1, visit 1; T2, visit 2; grand mean, mean from T1 and T2; MS_B_, mean square between; ICC, intraclass correlation coefficient; SEM, standard error of the measurement; CV, coefficient of variation; *P*‐value, type‐I error rate for the one‐way ANOVA used to assess systematic variability; slope 1, oxygen desaturation rate; $S_{{\mathrm{tO}}_{2}\min}$, minimum oxygen saturation observed; $S_{{\mathrm{tO}}_{2}\mathrm{imag}}$, magnitude of ischaemia; $S_{{\mathrm{tO}}_{2}\max}$, maximum oxygen saturation observed; slope 2, oxygen reperfusion rate; slope 2_10‐s_, oxygen reperfusion rate during the 10‐s window immediately post‐cuff deflation; $S_{{\mathrm{tO}}_{2}\mathrm{rmag}}$, reperfusion magnitude of oxygen saturation; time to max $S_{{\mathrm{tO}}_{2}}$, difference in time from $S_{{\mathrm{tO}}_{2}\min}$ to $S_{{\mathrm{tO}}_{2}\max}$; $S_{{\mathrm{tO}}_{2}\mathrm{AUC}}$, 2‐min reperfusion area under the curve after signal returned to baseline after cuff release.

**TABLE 4:** Test–retest reliability statistics for the variables derived from the near‐infrared spectroscopy with vascular occlusion test in a group of women (*n* = 30)

	T1 mean ± SD	T2 mean ± SD	Grand mean ± SD	MS_B_	MS_B_ (%)	ICC_2,1_ (95% CI)	SEM	CV (%)	*P‐*value
Slope 1 (%/s)	−0.105 ± 0.033	−0.111 ± 0.034	−0.108 ± 0.031	0.002	1.9	0.700 (0.463, 0.844)	0.02	16.6	0.317
$S_{{\mathrm{tO}}_{2}\min}$ (%$S_{{\mathrm{tO}}_{2}}$)	31.7 ± 12.2	29.8 ± 12.2	30.7 ± 11.4	259.3	843	0.742 (0.529, 0.868)	6.2	20.1	0.262
$S_{{\mathrm{tO}}_{2}\mathrm{imag}}$ (%$S_{{\mathrm{tO}}_{2}}$)	30.0 ± 9.1	31.3 ± 9.3	30.7 ± 8.4	141.5	461	0.672 (0.419, 0.828)	5.3	17.2	0.331
$S_{{\mathrm{tO}}_{2}\max}$ (%$S_{{\mathrm{tO}}_{2}}$)	74.1 ± 4.7	73.5 ± 4.8	73.8 ± 4.6	41.7	56	0.867 (0.742, 0.934)	1.7	2.3	0.221
Slope 2 (%/s)	1.032 ± 0.39	1.042 ± 0.35	1.037 ± 0.34	0.23	22	0.711 (0.474, 0.852)	0.2	19.3	0.845
Slope 2_10‐s_ (%/s)	1.463 ± 0.57	1.421 ± 0.64	1.442 ± 0.54	0.64	47	0.485 (0.151, 0.718)	0.5	34.7	0.930
$S_{{\mathrm{tO}}_{2}\mathrm{rmag}}$ (%$S_{{\mathrm{tO}}_{2}}$)	42.4 ± 12.0	43.7 ± 11.7	43.1 ± 11.0	244.1	467	0.734 (0.515, 0.864)	6.1	14.3	0.430
Time to max $S_{{\mathrm{tO}}_{2}}$ (s)	43.6 ± 11.2	43.8 ± 11.4	43.7 ± 10.5	220.2	504	0.737 (0.515, 0.866)	5.9	13.4	0.860
$S_{{\mathrm{tO}}_{2}\mathrm{AUC}}$ (%/s)	1114.9 ± 371.8	1073.6 ± 358.0	1094.2 ± 336.1	225992.6	20653	0.699 (0.460, 0.844)	200.9	18.4	0.433

Abbreviations: T1, visit 1; T2, visit 2; grand mean, mean from T1 and T2; MS_B_, mean square between; ICC, intraclass correlation coefficient; SEM, standard error of the measurement; CV, coefficient of variation; *P*‐value, type‐I error rate for the one‐way ANOVA used to assess systematic variability; slope 1, oxygen desaturation rate; $S_{{\mathrm{tO}}_{2}\min}$, minimum oxygen saturation observed; $S_{{\mathrm{tO}}_{2}\mathrm{imag}}$, magnitude of ischaemia; $S_{{\mathrm{tO}}_{2}\max}$, maximum oxygen saturation observed; slope 2, oxygen reperfusion rate; slope 2_10‐s_, oxygen reperfusion rate during the 10‐s window immediately post‐cuff deflation; $S_{{\mathrm{tO}}_{2}\mathrm{rmag}}$, reperfusion magnitude of oxygen saturation; time to max $S_{{\mathrm{tO}}_{2}}$, difference in time from $S_{{\mathrm{tO}}_{2}\min}$ to $S_{{\mathrm{tO}}_{2}\max}$; $S_{{\mathrm{tO}}_{2}\mathrm{AUC}}$, 2‐min reperfusion area under the curve after signal returned to baseline after cuff release.

In general, relative and absolute reliability was better in YW than MAOW, with the exception of $S_{{\mathrm{tO}}_{2}\max}$, which displayed marginally higher absolute and relative reliability in MAOW (CV = 2.0%; ICC_2,1_ = 0.87) versus YW (CV = 2.4%; ICC_2,1_ = 0.83). Finally, as indicated by the lack of differences between visit 1 and visit 2 (*P*‐values from the ANOVA table all >0.05), no systematic variability was observed for any measure.

### 3.2 Group differences

Baseline characteristics are displayed in Table 1, and vascular and metabolic outcomes are shown in Table 5 for YW versus MAOW. Overall, $S_{{\mathrm{tO}}_{2}\max}$, $S_{{\mathrm{tO}}_{2}\mathrm{imag}}$, $S_{{\mathrm{tO}}_{2}\mathrm{rmag}}$ and FMD% were significantly higher in the YW versus MAOW group (all *P* < 0.033), and slope 1, F_VA_, 10‐year risk (%) and MAP were significantly higher in the MAOW versus YW group (all *P* < 0.033). There were no age‐related differences in baseline $S_{{\mathrm{tO}}_{2}}$, slope 2, slope 2_10‐s_, time to max $S_{{\mathrm{tO}}_{2}}$, $S_{{\mathrm{tO}}_{2}\min}$ or $S_{{\mathrm{tO}}_{2}\mathrm{AUC}}$.

**TABLE 5:** Vascular and metabolic outcomes in younger versus middle‐aged and older women

Variable	YW (*n* = 17)	MAOW (*n* = 17)	*P*‐value
Baseline (%$S_{{\mathrm{tO}}_{2}}$)	62.8 ± 6.2	60.4 ± 4.3	0.185
Slope 1 (%/s)	−0.118 ± 0.031	−0.097 ± 0.025	**0.033**
$S_{{\mathrm{tO}}_{2}\min}$ (%$S_{{\mathrm{tO}}_{2}}$)	29.4 ± 13.9	33.1 ± 8.8	0.364
$S_{{\mathrm{tO}}_{2}\mathrm{imag}}$ (%$S_{{\mathrm{tO}}_{2}}$)	33.4 ± 8.9	27.3 ± 6.1	**0.025**
$S_{{\mathrm{tO}}_{2}\max}$ (%$S_{{\mathrm{tO}}_{2}}$)	75.8 ± 4.9	71.8 ± 3.8	**0.011**
Slope 2 (%/s)	1.026 ± 0.40	0.980 ± 0.29	0.698
Slope 2_10‐s_ (%/s)	1.425 ± 0.62	1.420 ± 0.43	0.980
$S_{{\mathrm{tO}}_{2}\mathrm{rmag}}$ (%$S_{{\mathrm{tO}}_{2}}$)	46.4 ± 11.4	38.7 ± 8.6	**0.033**
Time to max $S_{{\mathrm{tO}}_{2}}$ (s)	49.0 ± 12.8	41.5 ± 9.9	0.064
$S_{{\mathrm{tO}}_{2}\mathrm{AUC}}$ (%/s)	1168.9 ± 313.9	999.9 ± 309.1	0.124
FMD%	5.73 ± 2.10	3.87 ± 2.57	**0.028**
MAP (mmHg)	84 ± 10	92 ± 14	**0.026**

Values are calculated as the mean from T1 and T2 for each participant and are presented as group means ± SD. Four of the 34 participants were missing either T1 or T2 values. In these cases, their T1 or T2 values were used when calculating group means. Independent samples *t*‐tests were performed to identify group differences and resulting *P*‐values are displayed here with values shown in bold indicating statistically significant differences. YW, young women; MAOW, middle‐aged and older women; Slope 1, oxygen desaturation rate; $S_{{\mathrm{tO}}_{2}\min}$, minimum oxygen saturation observed; $S_{{\mathrm{tO}}_{2}\mathrm{imag}}$, magnitude of ischaemia; $S_{{\mathrm{tO}}_{2}\max}$, maximum oxygen saturation observed; slope 2, oxygen reperfusion rate; slope 2_10‐s_, oxygen reperfusion rate during the 10‐s window immediately post cuff deflation; $S_{{\mathrm{tO}}_{2}\mathrm{rmag}}$, reperfusion magnitude of oxygen saturation; time to max $S_{{\mathrm{tO}}_{2}}$, difference in time from $S_{{\mathrm{tO}}_{2}\min}$ to $S_{{\mathrm{tO}}_{2}\max}$; $S_{{\mathrm{tO}}_{2}\mathrm{AUC}}$, 2‐min reperfusion area under the curve after signal returned to baseline after cuff release; FMD%, percentage flow‐mediated dilatation; MAP, mean arterial pressure.

### 3.3 Relationships

**FIGURE 2:**
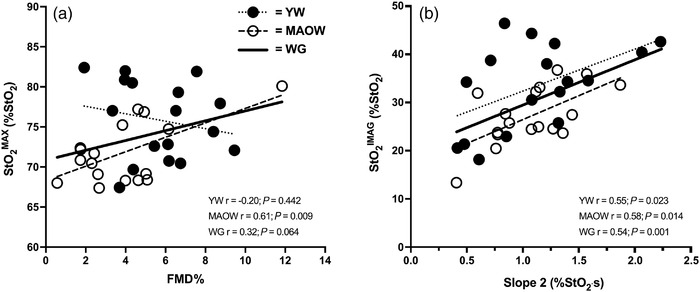
Associations between FMD% and $S_{{\mathrm{tO}}_{2}\max}$ (a) and slope 2 and $S_{{\mathrm{tO}}_{2}\mathrm{imag}}$ (b) in the WG (*n* = 34), and within the YW (*n* = 17) and MAOW (*n* = 17) groups. FMD%, percentage flow‐mediated dilatation; MAOW, middle‐aged and older women; $S_{{\mathrm{tO}}_{2}\mathrm{imag}}$, tissue ischaemic index, calculated as baseline minus minimum tissue oxygenation; $S_{{\mathrm{tO}}_{2}\max}$, maximum tissue oxygen saturation; WG, whole group; YW, younger women

Slope 2 was significantly related to $S_{{\mathrm{tO}}_{2}\mathrm{imag}}$ in the whole group (*r* = 0.54; *P* = 0.001), with similar relationships observed in both MAOW (*r* = 0.58; *P* = 0.014) and YW (*r* = 0.55; *P* = 0.023). No relationships were observed between $S_{{\mathrm{tO}}_{2}\mathrm{imag}}$ and FMD% in the whole group, in MAOW or in YW (*r* ≤ 0.33; *P* ≥ 0.192). $S_{{\mathrm{tO}}_{2}\max}$ was significantly related to FMD% in MAOW (*r* = 0.61; *P* = 0.009) but not in the YW (*r* = −0.20; *P* = 0.442), and therefore this relationship did not reach significance at the whole‐group level (*r* = 0.32; *P* = 0.064). These relationships are presented in Figure 2.

## 4 DISCUSSION

The primary purpose of this study was to examine the test–retest reliability of commonly reported microvascular function and metabolic parameters assessed using the NIRS‐VOT in YW and MAOW. A secondary purpose was to assess age‐related differences in these NIRS‐VOT‐derived variables in women. The main findings of this study were that: (1) the majority of NIRS‐VOT variables demonstrated moderate‐to‐good relative reliability (ICCs ≥0.51 and ≤0.87) with absolute reliabilities (CV%) of 17 ± 7%, where YW generally displayed greater reliability than MAOW; and (2) that YW displayed greater skeletal muscle O_2_ utilization and reperfusion magnitude than MAOW, whereas microvascular reactivity was not different. Overall, our data suggest that the NIRS‐VOT is a reliable, sensitive, simple, non‐invasive technique for the assessment of skeletal muscle metabolic and microvascular characteristics in both YW and MAOW. These preliminary findings have important implications for the planning and development of future studies employing the NIRS‐VOT to study skeletal muscle metabolic and microvascular function in women, and also provide important insights into the effects of age on these parameters in women.

### 4.1 Reliability

There are few, if any, data available which speak to the absolute and relative reliability of the NIRS‐VOT to study skeletal muscle metabolic and microvascular function in women specifically – a critically important gap given that there are sex‐specific reproductive and ageing effects on metabolic and vascular function. Our results suggest that most of the parameters derived from the NIRS‐VOT exhibit adequate reliability. Of these, the most commonly reported, which include slope 1, slope 2, $S_{{\mathrm{tO}}_{2}\mathrm{rmag}}$ and $S_{{\mathrm{tO}}_{2}\mathrm{AUC}}$, exhibited ICCs ranging between 0.74 and 0.79 with average CVs of 15 ± 3% among YW and ICCs ranging from 0.51 to 0.63 with CVs of 20 ± 2% among MAOW. Importantly, however, slope 2_10‐s_, which is likely a more specific indicator of microvascular reactivity than slope 2 (Barstow, 2019), demonstrated poor relative and absolute reliability in MAOW in this study. A few other studies have examined the reliability of parameters measured during the NIRS‐VOT, although these studies have primarily been in young men (Iannetta et al., 2019; Lacroix et al., 2012; McLay, Nederveen, et al., 2016). These studies have generally reported very similar ICCs and CVs to the present study for slope 2 and Slope 2_10‐s_ (except for slope 2_10‐s_ in MAOW) (Iannetta et al., 2019; Lacroix et al., 2012; McLay, Nederveen, et al., 2016) and for slope 1, $S_{{\mathrm{tO}}_{2}\max}$ and $S_{{\mathrm{tO}}_{2}\mathrm{AUC}}$ (Lacroix et al., 2012). Importantly, our study also examined reliability in a larger sample than in these prior studies, improving the accuracy of the ICC point estimates. Therefore, overall, our data suggest that the parameters obtained from the NIRS‐VOT are sufficiently reliable in women independent of age, except for Slope 2_10‐s_, $S_{{\mathrm{tO}}_{2}\mathrm{imag}}$ and Time to max $S_{{\mathrm{tO}}_{2}}$, which demonstrate poor reliability in MAOW.

### 4.2 Age‐related differences

To date, only one other study has examined age‐related differences in skeletal muscle metabolic and microvascular function using the NIRS‐VOT in women (Horiuchi & Okita, 2020). We hypothesized that we would observe steeper desaturation slopes (i.e., slope 1) and greater reperfusion rates (i.e., slope 2) in YW than MAOW, indicating greater skeletal muscle oxygen consumption and greater microvascular reactivity, respectively. Consistent with our hypothesis, slope 1 was 22% lower in YW than MAOW, indicating a greater metabolic rate during ischaemia in YW. However, contrary to our hypothesis, we did not observe differences in slope 2 or slope 2_10‐s_. Thus, our data suggest that there are no age‐related differences in skeletal muscle microvascular reactivity in otherwise healthy YW and MAOW.

Despite the greater rates of desaturation (i.e., slope 1) and the larger difference between baseline and $S_{{\mathrm{tO}}_{2}\min}$ (i.e., $S_{{\mathrm{tO}}_{2}\mathrm{imag}}$) observed in YW versus MAOW, there was no difference in $S_{{\mathrm{tO}}_{2}\min}$ between these groups (−11%; *P* = 0.364). Thus, the absolute tissue ischaemia experienced by YW and MAOW was not markedly different. Because the ischaemic insult (i.e., $S_{{\mathrm{tO}}_{2}\min}$) is the primary vasodilatory stimulus for the microvasculature (Rosenberry et al., 2018), the lack of apparent differences in microvascular reactivity observed between YW and MAOW in this study may be explained by the similar $S_{{\mathrm{tO}}_{2}\min}$ observed between age groups. Interestingly, several other studies examining the effects of age on microvascular reactivity using the NIRS‐VOT have also reported that slope 2 is similar between younger and older adults, especially among older adults who are otherwise healthy (de Oliveira et al., 2019; George et al., 2018; Horiuchi & Okita, 2020; Iannetta et al., 2019). Horiuchi & Okita (2020) also reported that $S_{{\mathrm{tO}}_{2}\min}$ during arterial occlusion is similar between YW and MAOW. In contrast, studies which assessed differences in slope 2 and the magnitude of ischaemia (i.e., $S_{{\mathrm{tO}}_{2}\min}$ or $S_{{\mathrm{tO}}_{2}\mathrm{imag}}$) in healthy young versus older adults with existing CVD risk factors found slope 2 and ischaemia to be greater in the younger adults (de Oliveira et al., 2019; Rosenberry et al., 2018). Moreover, when Rosenberry et al. (2018) standardized the ischaemic insult (i.e., $S_{{\mathrm{tO}}_{2}\min}$) between the younger and older adults, the age‐related differences in microvascular reactivity, as well as the hyperaemic area under the curve, were eliminated. George et al. (2018) reported that higher aerobic fitness is associated with greater microvascular reperfusion rates independent of age, suggesting that lower fitness explains lower microvascular reactivity in the context of ageing. Taken together, these results imply that skeletal muscle oxidative capacity, and subsequently the absolute ischaemia experienced during vascular occlusion, is a primary determinant of microvascular reactivity as measured during the NIRS‐VOT. Therefore, our interpretation is that the MAOW in this study experienced similar absolute ischaemia during the occlusion period and thus demonstrated a preserved skeletal muscle microvascular reactivity.

Although we observed no differences in microvascular reactivity, we did observe differences in the total reperfusion magnitude ($S_{{\mathrm{tO}}_{2}\mathrm{rmag}}$) and maximal tissue saturation ($S_{{\mathrm{tO}}_{2}\max}$), which were greater in YW than MAOW. As previously noted, a clear distinction between ‘reactive’ hyperaemia and ‘responsive’ hyperaemia should be drawn, whereby reactive hyperaemia should only represent the ability of the microvasculature to dilate in response to acute ischaemia (Rosenberry & Nelson, 2020). This reactive reperfusion period is estimated to last <30 s after cuff deflation, and therefore measurements such as slope 2 and slope 2_10‐s_ provide particular insight into the reactive phase of reperfusion. On the other hand, responsive hyperaemia is exaggerated reperfusion that is mediated or sustained by factors ensuing, or because of, the initial blood flow surge (Evanoff et al., 2016; Rosenberry & Nelson, 2020; Tucker et al., 2020). For example, microvascular dilatation necessitates an immediate increase in blood flow (Rosenberry & Nelson, 2020) and subsequently causes an increase in shear rate along the blood vessels of the limb, which represents the stimulus for arterial dilatation that accommodates sustained increases in overall blood flow. Therefore, reactive hyperaemia measurements that include assessments of hyperaemia beyond the initial 30 s may also reflect information regarding sustained responsive hyperaemic responses that include conduit artery vasodilatation and the secondary increase in overall flow (Corretti et al., 2002; Ducharme et al., 1999; Rosenberry & Nelson, 2020; Soares et al., 2020; Thijssen et al., 2011). Consequently, it is highly plausible that the differences in $S_{{\mathrm{tO}}_{2}\mathrm{rmag}}$ and $S_{{\mathrm{tO}}_{2}\max}$ suggest age‐related differences in ‘responsive’ hyperaemia between YW and MAOW, potentially due to differences in endothelial function of the conduit artery. In support of this hypothesis, and in‐line with previous findings (Jensen‐Urstad & Johansson, 2001), FMD% was lower in MAOW than YW (3.87% vs. 5.73%; *P* = 0.028). Furthermore, we observed a significant relationship between $S_{{\mathrm{tO}}_{2}\max}$ and FMD% in MAOW (*r* = 0.61; *P* = 0.009), which was not present in YW (*r* = −0.20; *P* = 0.442) and was weaker in the whole sample (*r* = 0.32; *P* = 0.064). Overall, these findings support the suggestion that $S_{{\mathrm{tO}}_{2}\max}$ may provide important information regarding the factors that regulate responsive hyperaemia and may imply that impaired conduit artery vasodilatation is limiting to responsive hyperaemia in MAOW but not YW. Taken together with our finding of preserved microvascular reactivity, these data may also suggest mechanistically distinct age‐related decrements in vascular function in women (e.g., NO bioavailability versus prostacyclin‐, adenosine‐ or endothelial cell hyperpolarization‐mediated vasodilatation). Future studies are needed to systematically test these hypotheses and also to determine the prognostic utility of parameters such as $S_{{\mathrm{tO}}_{2}\max}$ and $S_{{\mathrm{tO}}_{2}\mathrm{rmag}}$.

### 4.3 Limitations

Our study has several limitations. First, we were not able to assess the causal temporal link between age and our outcomes due to the limitations of our cross‐sectional design. Second, we did not measure adipose tissue thickness, which may attenuate absolute NIRS signals. However, the $S_{{\mathrm{tO}}_{2}}$% signal used herein is relative, representing the proportion of oxygenated haem to total haem, both of which are similarly influenced by adipose tissue (i.e., the numerator and the denominator are influenced to the same degree). Thus, it may not be necessary to correct for adipose tissue thickness when using $S_{{\mathrm{tO}}_{2}}$% (Barstow, 2019), and it would seem unlikely that differences in adipose tissue thickness would have differentially affected the signal in YW and MAOW in the current study. In addition, melanin concentrations, which were not controlled for in this study, likely influence the NIRS signal (Matas et al., 2002). However, as each participant served as their own control in reliability analyses, it is unlikely differences in melanin among our participants influenced these findings. Finally, while we did collect data on upstream vascular function (i.e., FMD%), our study was not designed to systematically determine the upstream mechanisms that may contribute to the observed impairments in skeletal muscle microvascular function because that was not the primary purpose of this study. Still, this study provides important insights and our data are hypothesis‐generating. To better evaluate these relationships and gain greater insight into the influences of conduit artery on skeletal muscle microvascular responsive hyperaemia, future studies should consider utilizing the simultaneous NIRS‐VOT and FMD test (Rasica et al., 2022), which allows both assessments to be collected in the same limb at the same time. Doing so will allow for direct examination of the effects of tissue ischaemia on microvascular reactivity and conduit artery shear rate, shear rate on FMD and FMD on microvascular responsive hyperaemia in the context of ageing.

### 4.4 Conclusion

In conclusion, the preliminary findings from the current study support the use of the NIRS‐VOT as a simple, reliable, non‐invasive technique for the assessment of peripheral skeletal muscle metabolic and microvascular function in both YW and MAOW. However, investigators should be aware that reliability of the parameters measured in the NIRS‐VOT tends to be lower in MAOW, and so studies employing repeated measures designs will want to consider this when determining sample sizes. Our data also demonstrate that muscle oxygen utilization rates (slope 1) and responsive hyperaemia ($S_{{\mathrm{tO}}_{2}\max}$, $S_{{\mathrm{tO}}_{2}\mathrm{rmag}}$) are adversely affected by ageing in women, which may be related to reduced conduit artery function. We also found that ischaemic index was greater in YW than in MAOW. Finally, our findings seem to suggest that MAOW have a preserved microvascular reactivity to a given ischaemic stimulus, perhaps suggesting that the mechanisms that specifically mediate reactive hyperaemia are preserved in otherwise healthy and active MAOW.

## AUTHOR CONTRIBUTIONS

The study was planned by Emily M. Rogers and Nathaniel D.M. Jenkins. Data collection was completed by Emily M. Rogers and Nile F. Banks. Writing and editing were performed by all authors listed. All authors approved the final version of the manuscript and agree to be accountable for all aspects of the work in ensuring that questions related to the accuracy or integrity of any part of the work are appropriately investigated and resolved. All persons designated as authors qualify for authorship, and all those who qualify for authorship are listed.

## CONFLICT OF INTEREST

E.M.R. and N.F.B. have received graduate assistant stipend funding from Woodbolt, LLC. E.M.R. has received grant funding from the American College of Sports Medicine. N.F.B. and N.D.M.J. have received grant funding from the National Strength and Conditioning Association. N.D.M.J. has received grant funding from the American Heart Association, the National Institutes of Health, Woodbolt, LLC, and Applied Food Sciences, Inc. and has received an NIH Clinical Research Loan Repayment Award.

## FUNDING

The current study did not receive any external funding. All costs were covered using N.D.M.J.’s start‐up funds.

## Supporting information

Statistical Summary Document

## Data Availability

The data will be made available by the first author upon request.
